# A System of Medicine

**Published:** 1911-09

**Authors:** 


**A System of Medicine.** By Many Writers. Edited by Sir CLIFFORD ALLBUTT, K.C.B., and HUMPHRY DAVY ROLLESTON, M.D., F.R.C.P. Vol. VIII. *Diseases of the Brain and Mental Diseases.* 2nd Edition. Pp. xii, 1,070. London: Macmillan and Co. Limited. 1910.

This volume differs in several respects from the corresponding volume of the first edition. Diseases of the skin, which were formerly described in this volume, have been left for a further concluding one.

New articles have been written by Dr. F. E. Batten on Acute Polioencephalitis, by Professor J. Michell Clarke on Recurrent Paralysis, and by Dr. James Collier on Apraxia and Agnosia. Dr. Bastian's article on Aphasia has been revised by Dr. James Collier, while Dr. Head has rewritten the account of the Occupation Neuroses. Dr. Batten has also written a new article on all the forms of Meningitis, with the exception of the tuberculous.

The work is begun by an article on the Experimental Pathology of the Cerebral Circulation, by Dr. Leonard Hill. This is followed by a very excellent and admirably compressed article by Sir David Ferrier on the Regional Diagnosis of Cerebral Disease.

In reviewing a work of this size it is impossible to describe, or indeed even to enumerate all the articles, each of which is written by an authority on his subject.

The latter part of the book is devoted to mental diseases. To this Dr. Savage writes the introduction, in which he discusses the various causes of insanity. Other important articles are written by Dr. Mercier on Vice, Crime and Insanity; by Drs. Shuttleworth and Fletcher Beach on Idiocy and Imbecility; and on the Epochal Insanities by Dr. Clouston. Among many other very excellent articles may be mentioned one by Dr. Conolly Norman on Systematised Delusional Insanity, and an interesting but short contribution by Dr. Milne Bramwell on Hypnotism. It need perhaps scarcely be added that this edition has been brought thoroughly up to date throughout.

